# Evaluation of a special needs dental workshop for health professionals and students in Trinidad and Tobago

**DOI:** 10.3389/froh.2022.951165

**Published:** 2022-12-02

**Authors:** Ramaa Balkaran, Maureen Perry, Anushka Maharaj, Amrita Rajhbeharrysingh, Jorma I. Virtanen

**Affiliations:** ^1^ School of Dentistry, The University of the West Indies St. Augustine, St. Augustine, Trinidad and Tobago; ^2^ Institute of Dentistry, University of Turku, Turku, Finland; ^3^ Arizona School of Dentistry & Oral Health, A.T. Still University, Mesa Arizona, United States; ^4^ Faculty of Medicine, University of Bergen, Bergen, Norway

**Keywords:** evaluation, health professional, dental education, special care dentistry, dental student education

## Abstract

**Aims:**

This study aimed to evaluate a special needs dental workshop for dentists, allied dental health professionals and students in Trinidad and Tobago.

**Methods:**

This feedback study conducted in 2019–2020, included two surveys, one at the time of the workshop and a second survey one year after it. The first survey utilized an anonymous self-administered questionnaire enquiring about the reason for attendance, profession/education, demographics, and difficulties faced in treating patients with special needs. The follow-up study used an online survey tool assessing the workshop and queried comments/suggestions. The Chi-square test served for statistical analysis.

**Results:**

Of the 176 attendees 131 participated (response: 74.4%). The majority (81.5%) were females. Most attendees were dental students (50.3%) or dentists (38.9%). Knowledge acquisition (73.1%) and professional development (14.2%) were the main motives for attendance while communication (31.8%) and non-compliance (34.5%) were the main problems faced in treating patients with special needs. The follow-up evaluation (post-workshop) (response: 72.5%) showed that most participants (90.5%) assessed the workshop as positive; 80% reported an increase in their knowledge and 64.2% perceived a need for further education.

**Conclusion:**

These findings suggest a considerable demand for special needs dental services and continuing education and show that workshops can actively affect patient care.

## Introduction

People with special needs may include those with disabilities such as impairments, activity limitations, and participation restrictions (1). People with special needs are at an increased likelihood of oral diseases (2). Developments in the health sector and care have led to a reduction in mortality rate, thus an increase in the elderly population of persons with disabilities, living with chronic health conditions (3). The Royal College of Surgeons of England has defined Special Care Dentistry (SCD) as the specialty of dentistry that “provides preventive and treatment oral care services for people who are unable to accept routine dental care because of some physical, intellectual, medical, emotional, sensory, mental or social impairment or a combination of these factors” (4). It is a new dental specialty worldwide and the term Special Needs Dentistry (SND) is also synonymous with SCD in some parts of the world. This field of Dentistry modifies treatment to treat these patients by promoting preventative dentistry and modifying routine dental care to adapt to specific patients' needs. Given the complexity involved in the management of persons with special needs, current research has shown that both the service provided and, the personnel may affect access to oral health care (5).

Recent research has shown several barriers that caregivers, as well as dental practitioners, encounter when treating patients with special needs (2). These barriers include limited practitioner experience in treating patients with disabilities, communication issues, and patient non-compliance with dental treatment. In some cases, a dentist inexperienced in this field may find it necessary to refer the patient to a specialist. This inevitably leads to a barrier in access to dental care since the patient will be turned away or may have to venture to another dentist for treatment.

Furthermore, from an educational perspective, it should be noted that persons with special needs may have developmental disabilities, complex medical problems, and significant physical limitations in addition to complex social situations. These issues may all lead to the modification of regular dental routines to provide dental treatment (6). Therefore, understanding the importance of providing quality dental care for patients with special needs is of extreme importance. Trinidad and Tobago are twin islands in the Caribbean. It has been estimated that approximately 4% (52,000) of the total population of 1.4 million persons, were living with a disability (7). In Trinidad and Tobago, there is a lack of accessibility to special needs dental care. Furthermore, very few dentists are specialized in this field locally and for those dentists that are interested in specializing, there are no local postgraduate opportunities, and those that exist internationally, require full-time training there. This leads to a greater demand for dental care in this population and dental practitioners must be capable of treating these patients. Based on preliminary findings from a clinical audit of the caregivers of persons with special needs, their experiences have largely been negative because most public healthcare facilities lack the resources, lack experience of the dentist and dental staff to adequately treat this population, and have physical accessibility issues (8).

Although dentists may be willing to treat patients with disabilities, they may lack the knowledge and/or tools to perform the treatment safely in either the public or private setting and may prefer to refer the patient to have the treatment done in a hospital setting. Additionally, a recent study showed health care professionals experience multiple barriers when treating patients with special needs in the country (9). A need for change in the provision and access of dental services for people with special needs and education of healthcare professionals were warranted.

To the best of the authors' knowledge, there have been no studies conducted locally, and very few studies internationally, on the evaluation of an educational seminar on special needs dentistry. This is the first time there was a workshop locally, in this field of special needs dentistry. The objective of this study was to evaluate the special needs dental workshop for dentists, dental students, and allied health professionals, and its impact on the attendees one year later. The workshop explored the treatment and management of patients with special needs. We investigated the participants' motives for attending the workshop, common difficulties encountered in treating patients with special needs, and their perceived learning outcomes. We also investigated whether the workshop benefitted their provision of oral health treatment for patients with special needs one year after attendance.

## Materials and methods

This evaluation of the dental workshop was conducted at the University of the West Indies, St. Augustine Campus, Trinidad and Tobago, in 2019–2020. The educational evaluation included two surveys, one at the time of the workshop to assess the level of interest in this topic and a second survey one year after the event, to assess the impact of the workshop. The UWI Ethics Committee reviewed and approved the study involving human participants (CRECSA.0073/11/2019 and CREC-SA.0600/11/2020). Participation was voluntary and anonymous, and this was stated at the beginning of each survey. All participants gave their written informed consent, which was required for this study in line with the institutional requirements of the UWI Ethics Committee.

### Workshop

The seminar-based workshop in November 2019 discussed the treatment and management of patients with special needs and when specialist referral for management under sedation may be required. There was a Special Needs Dentistry seminar and a lecture on Developmental Behavioral Pediatrics at the workshop. The latter brought a medical perspective to the special needs dental workshop and emphasized the need for collaboration between physicians and dentists in the effective treatment of persons with disabilities. The featured dental lecture was a seminar-based one that stressed the need to improve the oral health of persons with disabilities and ultimately improve their quality of life. The professor incorporated video clips to demonstrate different techniques to the attendees at the workshop.

### Participants

A convenience sample was obtained from all 176 attendees (dentists, dental therapists, dental assistants, and dental students, years 1–5, of the school) at the workshop, who were invited, upon entry to the venue, to participate in the first survey. The workshop itself had been advertised on national television, and on social media by the dental students' association, the Dental Association, and Dental Council as well as *via* flyers. One year after the workshop a second questionnaire was sent electronically to those who participated in the initial survey (*n* = 131). A cross-sectional survey was initially conducted, that invited all attendees at the workshop, to complete an anonymous self-administered questionnaire at the workshop. The survey was guided by a review study (10) and modified according to the local context. No culturally appropriate validated tools were available for this first ever workshop. The survey comprised quantitative assessments of the demographics (gender, age, ethnicity, enrolled degree for students, and year of graduation for the dentists) of the attendees, the number of patients with special needs that are usually treated per week, and their reason for attendance. It also involved open-ended questions on the barriers encountered while treating patients with special needs and their reasons for attending the workshop. When distributing the questionnaires, instruction, and information about the objectives of the study were clarified by the researchers (AM, AB, RB) who had been trained and calibrated before the workshop. All participants were given questionnaires and asked to return them to the box at the registration table upon completion of the 1st survey. (There were no identifiers on the questionnaires). This was followed by a feedback survey conducted one year later and sent electronically, due to COVID-19 restrictions. All attendees of the workshop were invited to respond to the 2nd survey. The responses of the participants were not identifiable *via* the website. This survey was based on a previous study (11) and modified based on the local context. The follow-up study used an online survey tool that quantitatively assessed the respondents' demographics and employed a 5-point Likert scale to assess the participants' assessment of the workshop, the knowledge, and confidence gained by the information, and the application of that education. It also assessed the comments of the participants about the most rewarding aspects of their experience and suggestions on how to improve the workshop.

All quantitative data were analyzed using the statistical software package for social science (SPSS version 25 IBM, Armonk, NY USA). The Chi-square test served in statistical analysis. The qualitative data were grouped into codes under six themes. All similar themes from the open-ended questions in both surveys were combined and coded. Three researchers then individually coded these themes and used a follow-up peer debriefing session to generate themes.

For example, under the theme knowledge, these similar themes were combined “to prepare for clinical years”, “to learn how to improve treatment for patient”s comfort”, “to know how to be able to care and improve skills in this field” Under the theme communication “patient may have limited knowledge and understanding”, “difficulty with informed consent”, “patient is non-communicative” were combined. Subsequently, the frequency of these themes stated by participants was assessed.

### Variables and analysis

Socio-demographic: Age in the 1st questionnaire was regrouped into the same three categories that were used in the 2nd survey -< 25, 25–40, and >40 years. Ethnicity was regrouped into 3 categories for both surveys- Indo-Caribbean, Afro-Caribbean, and Other. The 2nd survey educational program was grouped into “students” which comprised both preclinical and clinical students and the 2nd group of health care professionals comprised dentists, dental assistants, and dental therapists. The responses to the open-ended questions such as the most rewarding aspects and comments of the workshop were coded based on similar themes and entered. The analysis was limited to descriptive statistics.

## Results

**Figure 1:**
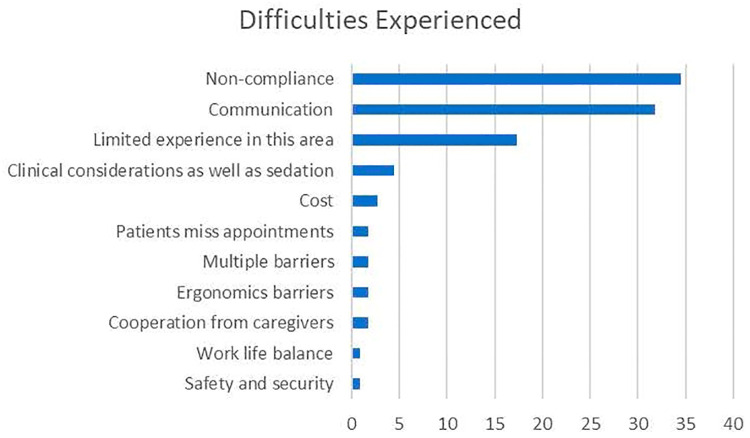
The most common themes on difficulties faced in treating patients with special needs.

Of the 176 attendees at the workshop, 131 took part in the first survey (response rate: 74.4%). Most of the respondents were females (81.5%) mean age of 27.6 (SD 7.41). Almost half (47.3%) were of Indo-Caribbean ethnicity. Most (89.2%) were dental students (50.3%) or dentists (38.9%) (Table 1). The most common themes for attendance at the workshop were knowledge (73.1%) followed by professional development (14.2%) (Table 2). The vast majority (68.2%) of respondents who saw patients with special needs reported that they saw 5 or less per week with just over one-fifth (21.7%) reported not seeing any such patients. Challenges expressed by the participants when treating patients with special needs included communication (31.8%), non-compliance (34.5%), and limited experience in this field (17.3%) (Figure 1).

**Table 1:** Demographics of participants in the two surveys.

Variables	*n* = 131 (Valid %)	*n* = 95 (Valid %)
	Survey I	Survey II
Gender		
Male	24 (18.3)	15 (15.8)
Female	106 (80.9)	80 (84.2)
Age		
Under 25	59 (45.7)	49 (51.6)
25-40	58 (44.3)	38 (40.0)
Over 40	12 (9.3)	8 (8.4)
Ethnicity		
Afro Caribbean	34 (26.2)	19 (20.0)
Indo Caribbean	62 (47.7)	48 (50.5)
Caucasian	1 (0.8)	0 (0)
Chinese	3 (2.3)	1 (1.1)
Mixed	27 (20.8)	23 (24.2)
Other	3 (2.3)	4 (4.2)
Educational Program		
Dentist	51 (38.9)	40 (42.1)
DSA/DHDT	14 (10.7)	7 (7.4)
Dental Student	80 (50.3)	48 (50.5)

**Table 2:** Participants’ reasons for attendance at the workshop - generated themes.

Reason for attendance	No. of Participants *n* = 131	Percentage of Participants
Knowledge	98	73.1
Professional Development	19	14.18
To improve clinical skills with respect to dealing with patients with special needs	13	9.7
Frequently treats patients with special needs	2	1.5
Family Members with special needs	1	0.75
Aid in Research	1	0.75

**Figure 2:**
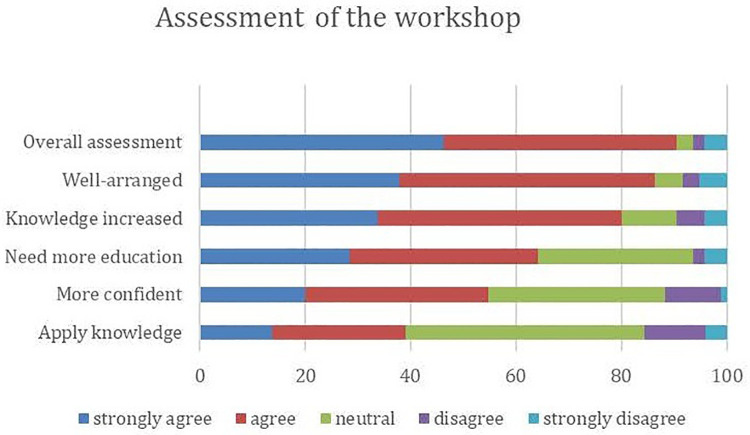
The participants’ assessment of the special needs workshop.

In the second survey, 95 of the 131 respondents participated which gave a response rate of 72.5%. The reason for this loss of sample of the participants who attended the workshop was not ascertained. Most participants (90.5%) had a positive view of the overall assessment of the workshop and 80% also responded positively that their knowledge increased and 64.2% felt that they needed more education (Figure 2). The comments from the participants were positive and the most rewarding aspects of the workshop included the experiences shared by the professor in special needs (Table 3).

**Table 3:** Suggestions from the attendees related to comments and rewarding aspects of the workshop^a^.

Comments	Most Rewarding aspects of the Workshop
Have more of these workshops (*n* = 7)	Getting a better insight of Prof Perry's experiences (*n* = 7)
Live recordings of special needs management in complicated circumstances can be a valuable teaching tool. (*n* = 2)	Gaining a better understanding in patient treatment (*n* = 4)
Introduce more techniques that can be used when treating special needs patients and how to better identify conditions (*n* = 2)	All parts of it were equally rewarding (*n* = 3)
More of these with foreigner lecturers (*n* = 2)	The results and the patients’ reactions (*n* = 2)
More insight on how to go about studying special needs dentistry. (*n* = 1)	Being able to interact in an open forum with the team (*n* = 2)
Even though a lot was shared, I think more information could have been covered. (*n* = 1)	Autism spectrum disorder management (*n* = 2)
Just that it was well organized and very insightful (*n* = 1)	The main lecture (*n* = 2)
It should be a series, multiple lectures over the course of some days with the whole team (*n* = 1)	Discussions about patients (*n* = 2)
Invite a special needs patient to shed some light on their perspective as a dental patient who has special needs (*n* = 1).	For the most part the questions being asked by participants (*n* = 1)
More practical teaching. (*n* = 1)	Explanation of past experiences (*n* = 1)
Give more information on complex medical conditions (*n* = 1)	The practicality of seeing how dentists deal with real patients with use of video (*n* = 1)
Have virtual ones (*n* = 1)	The successful experiences of dentists with their special needs patients (*n* = 1)
	ADA certificate (*n* = 1)

^a^ (in descending order of frequency) showing the themes chosen after coding the data.

## Discussion

This study presented insight into the evaluation of the first special needs dental workshop that was held for dental students of all years, dentists, and allied healthcare professionals in Trinidad and Tobago. There is one dental school in this country, since 1989, that hired the first lecturer in Special Needs Dentistry in 2018, although the school has had a clinic for persons with special needs since 2009. Most dentists in Trinidad and Tobago have attended this dental school for their undergraduate education. There is no option for postgraduate training in this field locally for the dental team and therefore the school provides the only source of training exposure to dentists to treat persons with special needs. Due to a lack of specialists in this field, persons with special needs, regardless of their age, seek dental treatment from pediatric dentists and general dentists who may offer sedation services. The dental school has a five-year dental program in which the first two years are pre-clinical followed by three clinical years and after graduation there is a one-year internship. Most participants received their education at this school and were thus younger compared to the general population of dentists in Trinidad and Tobago. Also, the workshop was advertised on television, *via* email, and social media, which may have been more accessible to the younger group and led to their attendance. The attendees were certainly interested in the topic since Trinidad and Tobago does not require continuing dental education. There was an overwhelmingly positive response to the workshop that showed a great need for continuous education in this field given that most of the participants mainly attended for knowledge, professional development, and improvement in their clinical skills. These foremost reasons for attendance may be a result of this field of special needs dentistry being a recently established specialty (12). Several studies have discussed the undergraduate curricula and how they can be modified to include this subject to improve the knowledge of dental students (13–15). However, internationally, there has still been a deficiency in the didactic and clinical curriculum of dental and medical students in this field and research has demonstrated the need to improve these areas for both these healthcare professionals (16).

The strength of this research is that it was the first evaluation study on this topic nationally and the results echoed international trends that there is minimal didactic and clinical experience in dental schools on the treatment of people with special needs (17). In this research project, it was noteworthy that there was indeed a sustained interest, whereby participants responded to the second survey, one year later. Staff involved in the dental treatment of persons with special needs are often very passionate about this field and are very much advocates for the improvement of care in this population. This was the first time a workshop was held on this topic locally and the persons who attended were those with a genuine interest in the field. This was evident in their responses to the reason for attendance. However, it was not possible to use a validated instrument to demonstrate how the course was perceived by the participants and locally suitable. In addition, due to the COVID restrictions at the time of the follow-up survey, a practical approach was found to be appropriate (Supplement 1 and 2).

Overall assessment of the workshop that was held in November 2019, was positive, however, concerning their confidence and clinical work, attendees were more cautious. This may have been due to the short duration of the one-day workshop, which was mainly based on lectures and not on extensive clinical training. Also, given the current pandemic of COVID-19, the dental school was closed for almost one year from March 2020 to January 2021 and most private dental clinics were mandated by the government of Trinidad and Tobago to only perform emergency dental work between the period March to June 2020 (18). This may have accounted for a reduction in the attendees’ ability to apply their knowledge from the workshop. However, the vast majority agreed that their knowledge improved following the workshop one year later and recent research has shown that new learning can have an impact on dental practice, based on the participant's ability to implement them (19). Additionally, the workshop was a seminar-based one and because dentistry is an experiential field that requires hands-on experience, participants may have required a clinical component to increase their application of knowledge and clinical skills.

On average, most of the participants saw less than five patients with special needs, weekly, even though the UWI dental school has a dedicated special needs dental clinic, and most of the participants were dental students. This low number of patients seen by the respondents may be a result of the respondents' inability to overcome difficulties experienced with the dental treatment of persons with special needs. Conversely, there may have been a low presentation of persons with special needs at the dental clinics, during the duration of this study. Our research found that non-compliance of patients with special needs was the most common challenge experienced in their dental treatment. This finding was similar to Vozza et al. (2016) report that the uncooperativeness of some patients affected dentists' willingness to treat people with special health care needs (20). It was also similar to recent local research where both healthcare professionals and caregivers shared similar experiences with dental care for this population (9). Moreover, participants stated that they had limited experience in this field, which may affect their confidence in treatment (21, 22). Al Saadi et al. (2018) reported that a lack of confidence in the undergraduate dental years leads to a reduction in the care of patients with special needs in practice (23). Additionally, a study by Shah et al. (2011) indicated that during training, dentists that had very little involvement with treating patients with special needs would rarely opt to treat those patients in their practice as opposed to those who had experience with such patients during training (11).

Research has shown that both positive attitudes and willingness of participants are important motivations for planning a continuing education (24). Furthermore, one year later, participants identified a few areas which can be useful in planning future workshops on special needs dentistry, not just locally but internationally. Most wanted more knowledge in special needs dentistry. Areas that could have been improved were having longer workshops, and more information on both the treatment of patients with special needs and patients with complex medical histories. The latter is consistent with research on the practice patterns in special care dentistry, in which the authors advocated for continuing education in older medically compromised patients, given that these patients were commonly seen in general private practice settings (25).

The workshop included the entire dental team, which is important in the treatment of patients with special needs. This was the first time a dental workshop included other team members, locally. It was important to include these allied health care professionals whose participation in the treatment of this population can serve to improve both their health care and access to dental treatment (26). There were practicing dental hygienists and therapists (DHDT) as well as dental assistants (DSA) in attendance and it should be noted that the UWI dental school had a DHDT program for 6 years, which concluded in the year this workshop was conducted. This may have accounted for their small numbers in attendance. The participants were of varying educational levels, with graduated dental participants who ranged in years of practicing experience from <1 year to 24 years which may also have accounted for a variety of curricula depending on when the graduate was taught. Modifications, therefore, need to be taught to dentists at both an undergraduate and postgraduate level through continuing education so that all dentists are equipped with the essential skill set needed to treat this population of patients. This will reduce the barriers that are currently faced by dentists. Moreover, there may be a need to further develop the current curriculum to ensure that it builds the capacity of the dental graduate in this field.

Another strength of this research is that based on the response of the participants at the evaluation, there was a sustained interest in this workshop one year later. This bodes well for future workshops in this field, and it may mean that the participants had a positive attitude towards the dental treatment of patients with special needs and may have an intention to treat patients with special needs which ultimately benefits this population, whose dental needs are usually underserved.

However, there were limitations in this study too. The reasons for non-response were not ascertained and the data was anonymous in both surveys; therefore, we were unable to compare individual participants' results, which limits our statistical analyses and introduces uncertainty. The lack of face-to-face interaction with the authors and participants due to the pandemic may also have reduced the response rate.

Additionally, the participants may have had increased personal commitments, given that this country had several restrictions in place, which constrained movement during the COVID-19 pandemic, which may also have limited the responses. The results should be cautiously extrapolated as this was a pilot study with a small sample in a single-center design. Also, the instruments used were not validated, as existing validated instruments were not culturally appropriate for assessing the awareness and interest of the participants in the field of special needs dentistry in Trinidad and Tobago.

## Conclusions

This study illustrates the need to further educate dental providers in Trinidad and Tobago in the field of special needs dentistry. In so doing, we hope that this will increase capacity through improved education and therefore improve access to dental treatment by persons with special needs.

## Data Availability

The raw data supporting the conclusions of this article will be made available by the authors, without undue reservation.
