# Supplementary material for: Evaluation of a special needs dental workshop for health professionals and students in Trinidad and Tobago
Source: Front Oral Health. 2022 Dec 2;3:951165. doi: 10.3389/froh.2022.951165 (PMC9766665; doi:10.3389/froh.2022.951165)
Supplement: Supplementary file 1 [file Datasheet1.pdf]

Pre workshop questionnaire- An assessment of the practitioners' interaction with patients with Special Needs

Answer all questions that apply

1. Age ________

2. Gender? Male ☐ Female ☐

3. What is your ethnicity?

   Afro Caribbean ☐
   Indo Caribbean ☐
   Caucasian ☐
   Chinese ☐
   Mixed ☐
   Other ☐

4. What program are you/ or were you studying?

   DHDT ☐ DDS ☐ DSA ☐ MBBS ☐

5. If you are a student, what year are you currently enrolled in at the dental school?

   Year 1 ☐ Year 2 ☐ Year 3 ☐ Year 4 ☐ Year 5 ☐ Intern/Resident ☐

6. If you are a graduate, what year did you graduate?

2019	2018	2017	2016	2015	2014	2013	2012	2011	2010
☐	☐	☐	☐	☐	☐	☐	☐	☐	☐
2009	2008	2007	2006	2005	2004	2003	2002	2001	2000
☐	☐	☐	☐	☐	☐	☐	☐	☐	☐
1999	1998	1997	1996	1995	1994				
☐	☐	☐	☐	☐	☐				

7. What is your main reason for attending today’s workshop?

____

____

____

8. How many patients with Special Needs do you treat in a week?

____

____

____

9. What difficulties do you face as a practitioner?

____

____

____
