# Supplementary material for: Evaluation of a special needs dental workshop for health professionals and students in Trinidad and Tobago
Source: Front Oral Health. 2022 Dec 2;3:951165. doi: 10.3389/froh.2022.951165 (PMC9766665; doi:10.3389/froh.2022.951165)
Supplement: Supplementary file 2 [file Datasheet2.pdf]

Post workshop questionnaire- An assessment of the practitioners' views on the Special Needs Workshop

Please answer all questions that apply

1. Age ________
2. Gender? Male ☐ Female ☐
3. What is your ethnicity?
   - Afro Caribbean ☐
   - Indo Caribbean ☐
   - Caucasian ☐
   - Chinese ☐
   - Mixed ☐
   - Other ☐
4. What program are you/ or were you studying?

   DHDT ☐ DDS ☐ DSA ☐ MBBS ☐

5. If you are a student, what year are you currently enrolled in at the dental school?

   Year 1 ☐ Year 2 ☐ Year 3 ☐ Year 4 ☐ Year 5 ☐ Intern/Resident ☐

6. If you are a graduate, what year did you graduate?

2019	2018	2017	2016	2015	2014	2013	2012	2011	2010
☐	☐	☐	☐	☐	☐	☐	☐	☐	☐
2009	2008	2007	2006	2005	2004	2003	2002	2001	2000
☐	☐	☐	☐	☐	☐	☐	☐	☐	☐
1999	1998	1997	1996	1995	1994				
☐	☐	☐	☐	☐	☐				

Please answer the following on the scale of:

1 strongly disagree ‖ 2 disagree ‖ 3 neutral ‖ 4 agree ‖ 5 strongly agree

7. My overall assessment of the Special Needs dentistry workshop was positive

1 ☐ 2 ☐ 3 ☐ 4 ☐ 5 ☐

8. The Special Needs dentistry workshop was well arranged

1 ☐ 2 ☐ 3 ☐ 4 ☐ 5 ☐

9. The professor was well prepared

1 ☐ 2 ☐ 3 ☐ 4 ☐ 5 ☐

10. The learning objectives of the Special Needs dentistry workshop were appropriate

1 ☐ 2 ☐ 3 ☐ 4 ☐ 5 ☐

11. The learning objectives of the Special Needs dentistry workshop were met

1 ☐ 2 ☐ 3 ☐ 4 ☐ 5 ☐

12. I will be able to apply these skills to my practice

1 ☐ 2 ☐ 3 ☐ 4 ☐ 5 ☐

13. The amount of time scheduled was exactly what was needed to meet the learning outcomes

1 ☐ 2 ☐ 3 ☐ 4 ☐ 5 ☐

14. The lecturer demonstrated thorough knowledge of Special Needs dentistry

1 ☐ 2 ☐ 3 ☐ 4 ☐ 5 ☐

15. My knowledge and/or skills on Special Needs dentistry has greatly increased as a result of this workshop

1 ☐ 2 ☐ 3 ☐ 4 ☐ 5 ☐

16. I would recommend this Special Needs dentistry workshop to colleagues

1 ☐ 2 ☐ 3 ☐ 4 ☐ 5 ☐

17. Which part of the Special Needs dentistry workshop did you find most interesting?

____

____

____

18. What part of the Special Needs dentistry workshop did you find needed to be improved and what suggestion(s) do you have?

____

____

____

19. Do you have any further comments on the Special Needs dentistry workshop?

____

____

____
